# Development of a Brief Instrument for Assessing Healthcare Employee Satisfaction in a Low-Income Setting

**DOI:** 10.1371/journal.pone.0079053

**Published:** 2013-11-05

**Authors:** Rachelle Alpern, Maureen E. Canavan, Jennifer T. Thompson, Zahirah McNatt, Dawit Tatek, Tessa Lindfield, Elizabeth H. Bradley

**Affiliations:** 1 University of North Carolina at Chapel Hill, Chapel Hill, North Carolina, United States of America; 2 Yale School of Public Health, New Haven, Connecticut, United States of America; 3 Department of Population Medicine, Harvard Pilgrim Health Care Institute, Boston, Massachusetts, United States of America; 4 Suffolk Primary Care Trust, Suffolk, England; National Taiwan University, Taiwan

## Abstract

**Background:**

Ethiopia is one of 57 countries identified by the World Health Report 2006 as having a severely limited number of health care professionals. In recognition of this shortage, the Ethiopian Federal Ministry of Health, through the Ethiopian Hospital Management Initiative, prioritized the need to improve retention of health care workers. Accordingly, we sought to develop the Satisfaction of Employees in Health Care (SEHC) survey for use in hospitals and health centers throughout Ethiopia.

**Methods:**

Literature reviews and cognitive interviews were used to generate a staff satisfaction survey for use in the Ethiopian healthcare setting. We pretested the survey in each of the six hospitals and four health centers across Ethiopia (98% response rate). We assessed content validity and convergent validity using factor analysis and examined reliability using the Cronbach alpha coefficients to assess internal consistency. The final survey was comprised of 18 questions about specific aspects of an individual's work and two overall staff satisfaction questions.

**Results:**

We found support for content validity, as data from the 18 responses factored into three factors, which we characterized as 1) relationship with management and supervisors, 2) job content, and 3) relationships with coworkers. Summary scores for two factors (relationship with management and supervisors and job content) were significantly associated (P-value, <0.001) with the two overall satisfaction items. Cronbach's alpha coefficients showed good to excellent internal consistency (Cronbach alpha coefficients >0.70) for the items in the three summary scores.

**Conclusions:**

The introduction of consistent and reliable measures of staff satisfaction is crucial to understand and improve employee retention rates, which threaten the successful achievement of the Millennium Development Goals in low-income countries. The use of the SEHC survey in Ethiopian healthcare facilities has ample leadership support, which is essential for addressing problems that reduce staff satisfaction and exacerbate excessive workforce shortages.

## Introduction

The severely limited number of health professionals in sub-Saharan Africa negatively affects all types of health outcomes and threatens to limit the attainability of the Millennium Development Goals. The World Health Report 2006 is dedicated to recognizing and addressing these workforce shortages. The report identified a total of 57 countries that had a critical shortage of healthcare employees with a global deficit of 2.4 million doctors, nurses, and midwives [1]. Ethiopia has one of the greatest shortages with a density of only 0.03 physicians, 0.23 clinical nurses, and 0.02 midwives per 1,000 people in 2010 [2]. Several areas of human resources have been linked with barriers to achieving the Millennium Development Goals including low morale and motivation of health care workers, poor policies and practices for human resource development, and lack of supportive supervision for health workers [3]. Although recruitment is critical for addressing the shortage, retaining existing workers and instituting a scale up of successful programs is equally central to address the workforce crisis.

The Ethiopian Federal Ministry of Health has recently emphasized the need to produce and retain more health workers, and increased efforts to improve human resource management in hospitals through the Ethiopian Hospital Management Initiative (EHMI) [4], [5]. Experts in human resource management recognize the significant relationship between poor staff satisfaction and lower employee retention rates, particularly in low-income countries [6]. Despite the importance of staff satisfaction for employee retention, measures of staff satisfaction in health care organizations in low-income countries are limited. We identified five instruments [6], [7], [8], [9], [10] that have been validated as effective measures of satisfaction or motivation levels of employees in the healthcare settings; however, none had been designed for healthcare employees including both nurses and non-nurses in low-income countries. Furthermore, no instrument existed for use specifically in Ethiopia.

Accordingly, we sought to develop and validate a staff satisfaction measurement instrument for use with health care workers in government hospital and health centers throughout Ethiopia. To accomplish this objective, we used stakeholder interviews and existing literature to develop sets of items and tested the instrument in ten healthcare facilities (six hospitals and four health centers), assessing both construct validity and internal consistency. The resulting instrument may be helpful in Ethiopia's ongoing efforts to expand the health workforce and strengthen its health system.

## Methods

### Ethics Statement

All research procedures were approved by the Institution Review Board of the Yale School of Medicine, the Ethiopian Ministry of Health, and the Centers for Disease Control. We obtained Human Investigation Committee (HIC) exemption (protocol number 1010007494) for our study which waived the need for participant consent because no identifying participant information was obtained. Additionally, all participants were provided with an information sheet to let them know what data would be collected, how it would be used and disseminated, and any risks that would be encountered by participation. The information sheet was translated and distributed to ensure that participants fully understood that involvement in the study was voluntary, they could refuse to participate at any time, and there were no penalties if they declined participation. In order to maintain participant anonymity, we provided envelopes and sealed collection boxes for employees to return completed surveys; their names and specific job titles were not recorded and the name of the health facility they worked at was kept strictly confidential.

### Survey Design

To develop the Satisfaction of Employees in Health Care (SEHC) survey, we first conducted a literature review to identify validated surveys used to measure staff satisfaction in healthcare settings. We found several pre-validated instruments used in various areas, and identified questions within these instruments that could be used to assess satisfaction of staff at all levels in low-income countries. We located five validated surveys from which we extracted relevant questions: the Job Satisfaction Survey [7], the Emergency Physician Job Satisfaction Instrument [8], the Measure of Job Satisfaction survey (designed for use in monitoring the morale of community nurses in four trusts) [9], Motivational Outcome Constructs and Questions (designed for use in district hospitals in Kenya) [10], and a job satisfaction scale developed to study the correlation between job satisfaction and turnover intent of primary healthcare nurses in rural South Africa [6]. All extracted questions had to be appropriately modified for the use of measuring job satisfaction of all levels of employees in the Ethiopian healthcare setting. We identified additional factors that should be included or excluded from our final survey using 11 remaining articles from our literature review that were relevant, but did not include validated surveys [11], [12], [13], [14], [15], [16], [17], [18], [19], [20], [21].

### Survey Translation

Individuals from the Medical Services Directorate (MSD) and the Clinton Health Access Initiative (CHAI) who were fluent in both English and Amharic translated the survey tool from English into Amharic, Oromifa, and Tigrinya. During this translation process, a committee was formed consisting of CHAI Ethiopian Hospital Management Initiative (EHMI) team members who have technical background in Hospital Management, a strong understanding of the goals of the survey, and strong bilingual skills across the four languages. A CHAI staff member made an initial translation for each question into each language, and then a different CHAI staff member made a back translation independently. At this point, the EHMI project team reviewed each back-translation. Last, project team members and translators met and discussed any discrepancies between the original questions and back-translations to agree upon the most appropriate final translation. Translation from English to Amharic was considered a priority, because Amharic is the official language of Ethiopia, used nationwide, and the official working language of the Federal Democratic Republic of Ethiopia. The EHMI project team member maintained a complete database on translation procedures, literature review findings, and survey refinement which was shared with both CHAI and MSD. Although this database was not released to the public, documented information on study procedures can be obtained by contacting the authors.

### Cognitive Interviews

After compiling and translating potential items, we performed cognitive interviews [22] to enhance the survey comprehensibility and applicability to all employees in hospitals and health centers. We conducted five cognitive interviews in two hospitals in Addis Ababa, Ethiopia. In order to ensure our questions were relevant to different types of workers we selected interviewees from a range of jobs within the hospital: Acting Medical Director, Health Management Information Systems (HMIS) Officer, Quality Management Team Officer, Physician, and Cleaner. During these interviews, we probed respondents about the comprehensibility and meaning of each item, with particular focus on the translation from English to Amharic. In addition, as recommended by experts in cognitive interviewing [22], [23], we asked each respondent to comment on the content of the survey, including whether important topics might be missing or whether any questions were irrelevant. We used the results of the cognitive interviews to revise the survey tool, including translation modifications, refining the items, dropping questions that were ambiguous and modifying others to be more understandable.

**Figure 1:**
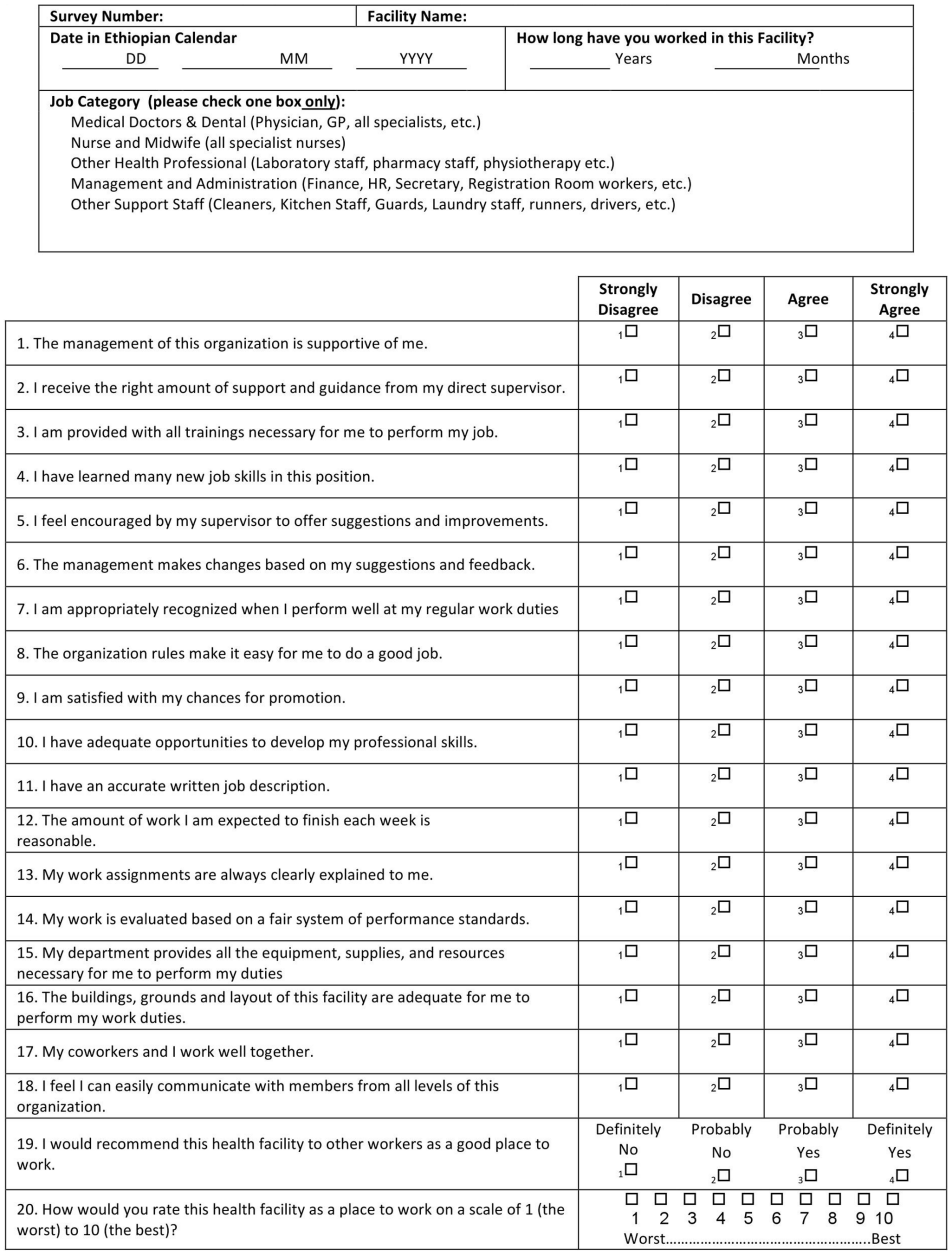
Satisfaction of Employees in Health Care (SEHC) Survey (*English*).

Before piloting, we distributed the survey among relevant stakeholders: the Ministry of Health, Black Lion Hospital (the largest hospital in the country), MSD and CHAI. Based on their suggestions, we made some adjustments, including eliminating, adding, and modifying questions and their order. The final survey was translated into three different languages (Amharic, Oromifa, and Tigrinya) and approved by MSD. Questions focused on relationships with management and supervisors, job content, and relationships with coworkers **(** **Figure 1** **)**.

### Pilot Testing

We piloted the survey tool in six hospitals and four health centers across four regions (Amhara, Oromia, SNNPR and Tigray) and one city administration (Addis Ababa) throughout Ethiopia, selected by Regional Coordinators from CHAI and MSD. The sample size of 70 surveys per facility was calculated conservatively, assuming at least 80% statistical power to detect a difference of 1.0 on the staff satisfaction 0–10 scale with a standard deviation of 1.8 and 95% confidence. Hence, we approached all staff at hospitals with less than 70 employees, at least 70 staff at hospitals with between 70 and 140 employees, and at least 50% of staff at hospitals with more than 140 employees. In facilities with greater than 70 workers, participants were randomly selected from the list of all employed clinical and technical staff, including those in management positions. In facilities with 70 or fewer workers a census of all clinical, technical and management employees was used. A total of 492 of the 500 staff approached (response rate 98%) completed face-to-face interviews with trained data collectors at both hospitals and health centers. At hospitals, project team members were assisted by the hospital staff member who was responsible for future SEHC survey distribution. Interpreters were not needed, as team members were fluent in the local languages. We added two additional questions to the survey for the pilot: a free response question asking whether there are any topics related to staff satisfaction that we did not ask about, and a fifth response column for asked if the response understood the question being asked to them. Survey data were double-entered into Microsoft Word templates by two different individuals to ensure data accuracy and then imported into Excel. Discrepancies between the two data entries were resolved by referring to the original paper survey. The excel file and a Microsoft Access database were distributed to all hospitals and health centers. We also provided detailed training on survey management and database usage, including principles of data entry, so that hospital staff could make the best use of these programs. Additional information can be obtained by contacting the authors.

Data collection took approximately four weeks, with one day spent collecting data at each site. Approximately 10 minutes was spent per participant explaining the study and obtaining their written consent for participation. Participants were asked to complete the survey independently; interviewers were available to read questions aloud if requested, though he/she was not allowed to answer any questions about the content. The survey was designed to require an average of 30 minutes to complete. Interviewers were trained in one day.

### Data Analysis

We evaluated construct validity, internal consistency, and convergent validity to assess the instrument. To assess construct validity, we conducted an varimax orthogonal rotated factor analysis with three factors specified *a priori* based on our hypotheses about distinct concepts (i.e., relationship with management & supervisors, job content, relationships with coworkers) and confirmed the number of factors using a Scree test [24]. Survey items that did not load strongly on any factor (i.e., loadings <0.30) or exhibited inadequate variability were dropped from further analysis. For each factor, we calculated the Cronbach's alpha coefficient to assess the internal consistency of the items with an alpha coefficient of 0.70 as the lower threshold for good reliability [25]. Additionally, we evaluated if any items, when removed, substantially changed the Cronbach's alpha coefficients. To assess convergent validity, we examined the association between each of the individual survey items and the two overall measures of staff satisfaction: “How would you rate this health facility as a place to work on a scale of 1 (the worst) to 10 (the best)?” and “I would recommend this health facility to other workers as a good place to work.” We created summary scores for each factor by summing responses of items that loaded on that factor and dividing by the number of responses, and then assessed the correlation between the three summary scores and each of the overall staff satisfaction items.

For all analyses, we used multiple imputation [26] to calculate estimates for the missing item responses, which comprised 10% or less for 93% of respondents. A total of 35 surveys (7% of respondents) had between 10% and 50% missing item responses. Multiple imputation procedure was performed using a series of imputed data sets created by running an imputation model that incorporated all 20 survey items. The analysis was replicated for each of the 10 imputed dataset and then the resulting estimates were compiled. We conducted the analysis with the full sample (n = 492) using imputation, as well as with the smaller sample of respondents (n = 457) with 10% or less missing item responses. Results were largely similar, thus we presented the findings from the full sample. We confirmed that imputed estimates matched valid answer values and were within the acceptable ranges for the 20 questions included in the final survey. All data analyses were performed using SAS V 9.2.

## Results

### Demographic Characteristics of Respondents

Of the 492 completed responses, 303 (61.6%) were from hospital employees, and 189 (38.4%) were from health center employees fulfilling a range of positions; the survey was distributed in a four different languages, Amharic, Oromifa, Tigrinya and English, in diverse regions of the country, and responders represented an assortment of positions in the facility **(** **Table 1** **)**.

**Table 1:** Demographic characteristics for staff participants (N = 492).

Variable	N (%)
**Facility Type**	
Hospital	303 (61.6%)
Health center	189 (38.4%)
**Language**	
Amharic	393 (79.9%)
English	7 (1.4%)
Oromifa	49 (10.0%)
Tigrinya	43 (8.7%)
**Geographical Region**	
Addis	189 (38.4%)
Amhara	99 (20.1%)
Oromia	101 (20.5%)
Southern Nations, Nationalities and People's Region (SSNPR)	50 (10.2%)
Tigray	53 (10.8%)
**Staff Position**	
Management and administration	86 (17.5%)
Medical doctors and dental	22 (4.5%)
Nurse and midwife	130 (26.4%)
Other health professional	94 (19.1%)
Other support staff	72 (14.6%)
Missing	88 (17.9%)

### Validity and Internal Consistency

The data supported three factors, or constructs, as hypothesized: (1) relationships with management and supervisors, (2) job content, and (3) relationships with coworkers. Results from the Scree test also suggested three distinct factors evidenced by the point at which the curve began to level off. Each retained item had a factor loading of 0.40 or higher on at least one of the factors and demonstrated strong construct validity **(** **Table 2** **)**. Three items loaded on both job content and relationships with management and supervisors factors **(** **Table 2** **)**. We dropped two items due to inadequate variability or loadings <0.40 for all factors. A third item was dropped because its removal resulted in significantly (P-value <0.05) increased Cronbach's alpha coefficient. These modifications resulted in a total of 18 survey items on specific aspects of employee experience plus two measures of overall staff satisfaction for a 20-item final survey instrument **(** **Figure 1** **)**. Internal consistency of the items in each of the three factors indicated good to excellent reliability (Cronbach alpha coefficients were 0.89, 0.70 and 0.70 for factors 1–3 respectively).

**Table 2:** Factor analysis of staff satisfaction survey item (N = 492).

	Factor 1	Factor 2	Factor 3	
	Relationship with Management & Supervisors	Job Content	Relationships with Coworkers	Original source reference*
***Factor Analysis*** *				
Q1. The management of this organization is supportive of me.	0.66	–	–	8, 10, 13, 15, 17, 18, 20, 21
Q2. I receive the right amount of support and guidance from my direct supervisor.	0.63	–	–	8, 10, 13, 15, 17, 18, 20, 21
Q3. I am provided with all trainings necessary for me to perform my job.	0.47	–	–	6, 7, 13, 15, 16, 17, 18, 20
Q4. I have learned many new job skills in this position.	0.45	–	–	6, 7, 13, 15, 16, 17, 18, 20
Q5. I feel encouraged by my supervisor to offer suggestions and improvements.	0.72	–	–	8, 10, 13, 15, 17, 18, 20, 21
Q6. The management makes changes based on my suggestions and feedback.	0.70	–	–	8, 10, 13, 15, 17, 18, 20, 21
Q7. I am appropriately recognized when I perform well at my regular work duties.	0.59	0.44	–	13, 16
Q8. The organization rules make it easy for me to do a good job.	0.50	0.40	–	10,21
Q10. I have adequate opportunities to develop my professional skills.	0.45	0.40	–	6, 7, 13, 15, 16, 17, 18, 20
Q13. My work assignments are always clearly explained to me.	0.46	–	–	21
Q14. My work is evaluated based on a fair system of performance standards.	0.43	–	–	21
Q9. I am satisfied with my chances for promotion.	–	0.41	–	6, 10, 13, 17
Q11. I have an accurate written job description.	–	0.46	–	21
Q12. The amount of work I am expected to finish each week is reasonable.	–	0.42	–	12, 14, 16
Q15. My department provides all the equipment, supplies, and resources necessary for me to perform my duties.	–	0.47	–	6, 7, 12, 13, 18
Q16. The buildings, grounds and layout of this health facility are adequate for me to perform my work duties.	–	0.55	–	7, 8, 15
Q17. My coworkers and I work well together.	–	–	0.65	8, 9, 10, 12, 15, 16, 18
Q18. I feel I can easily communicate with members from all levels of this organization.	–	–	0.60	21

* –‘ Indicates that questions have a factor loading of <0.4.

* Indicates the reference number for the original source of each SEHC survey question.

The associations between the summary scores for each of the factors and the overall staff satisfaction measures were statistically significant (all P-values <0.001) **(** **Table 3** **)**. The magnitude of the associations between overall staff satisfaction items and two of the summary scores (one measuring the employee's relationship with management and supervisors and one measuring job content) indicated moderate effect sizes (correlation coefficients ranged from 0.40 to 0.44) [25]. The association between overall staff satisfaction measures and the summary score measuring relationships with coworkers was statistically significant but of more modest in magnitude (correlation coefficients 0.14 and 0.15).

**Table 3:** Convergent validity: Correlation coefficients for scales with overall staff satisfaction measures (N = 492).

	Factor 1	Factor 2	Factor 3
	Relationship with Management & Supervisors	Job Content	Relationships with Coworkers
	Correlation Coefficient (p-value)	Correlation Coefficient (p-value)	Correlation Coefficient (p-value)
Q20. How would you rate this health facility as a place to work on a scale of 1 (the worst) to 10 (the best)?	0.43 (<0.001)	0.41 (<0.001)	0.14 (0.003)
Q19. I would recommend this health facility to other workers as a good place to work.	0.42 (<0.001)	0.40 (<0.001)	0.15 (0.001)

## Discussion

The SEHC survey, developed for use in Ethiopian health care facilities with diverse types of staff, was shown to have strong construct validity, excellent internal consistency and modest convergent validity. Although the instrument should be tested in additional low-income settings, these early data suggest this 20-item survey may be both practical and valid in assessing employee satisfaction in resource-limited hospitals and health centers in low-income countries like Ethiopia. As countries turn to improve the quality of health care and seek to recruit and retain a strong health workforce, the availability of practical tools to assess employee satisfaction is paramount. Data from such assessments can be important inputs to managerial decisions seeking to create organizational culture where staff flourish and provide high quality health services to people in need.

To our knowledge, SEHC is the first survey that successfully captures satisfaction across all levels of employees within both hospital and health clinic facilities in a low-income country. Of the five existing surveys from which we extracted and modified questions, the Job Satisfaction Survey [7] was most relevant, as it was designed to capture job satisfaction levels of all employees in an organization; however it was not designed specifically for the health care setting. Both the Emergency Physician Job Satisfaction Instrument [8] and the Measure of Job Satisfaction survey [9] were designed solely for the hospital setting, and only targeted specific populations (emergency physicians and community nurses). The job satisfaction scale to study the correlation between job satisfaction and turnover intent [6] was designed for use in rural South Africa but solely for nurses in clinics. Lastly, the Motivation Outcome Constructs and Questions [10] was useful, as it was also designed for use in hospitals in a similar developing country in Africa, but it was only intended to measure health worker motivation and not overall job satisfaction.

The SEHC survey represents a critical step in the pathway of addressing human resource issues necessary to help meet the Millennium Development Goals as well as a situation with characteristics necessary for a successful scale up. This survey has been shown to be reliable, valid and easy to implement within the hospital and health care setting to measure the satisfaction of all health care workers, a critical component for tools that are helpful in scale up [27]. Additionally, political support has been cited as an important component of effective scale-up framework [27], [28], [29], influencing the desirability for adoption and assimilation of an intervention [28], and has been noted to be an integral component for many successfully scaled up interventions in low- and middle-income countries [30], [31], [32], [33], [34]. The Ethiopian Ministry of Health demonstrated a leadership commitment to using the SEHC survey by adopting it into the national reformation guidelines and including a SEHC overall staff satisfaction score into the key performance indicators reported annually by hospitals to the government. Although this study shows implementation within one low-income country only, it demonstrates a process and tool that could be applicable to other low-income countries and suggests that future testing of this survey in other low-income countries is warranted to test the generalizability of the SEHC survey.

Several additional issues are important to recognize in order to promote the proper administration of the survey in the healthcare setting. Training in data collection and analysis is necessary. As briefly noted in the methods, our training program provided detailed instruction on survey management. We advised which staff members should be involved in the data collection process, we identified a human resources or quality improvement staff member to be responsible for arranging the survey distribution, and we ensured that a trained and independent individual was available to assist staff who could not read with survey completion. This person was trained to not attempt to explain or interpret questions, which might create bias, but merely to read the questions out loud. Additionally, a database in Microsoft Access was developed and distributed, which produces a final report that summarizes responses for each question. The database also has additional capabilities, such as comparing changes in both overall category averages as well as individual question averages within categories. Last, the training package included a brief introduction to the four stages of the Plan Do Study Act (PDSA) Cycle [35] as a method of introducing change based on the analysis of survey results [35], [36].

In addition, ensuring confidentiality of respondents is vital in order to encourage truthful responses by employees. In order to maintain confidentiality, we recommend that the data collector should provide envelopes in which completed surveys may be placed to maintain anonymity, as well as sealed collection boxes for employees to return their completed survey. Confidentiality should be explained to employees through promotional posters that also provide logistical details and encourage staff to complete the survey. We stressed that employees should never feel that they might be punished for what they report on a survey, and that participation is voluntary.

Last, specifying the minimum number of surveys to be collected from each facility should be informed by the statistical power needed to compare sites and analyze differences in single facilities over time. By ensuring that we collected large enough of a sample to reach at least 80% power, we feel confident that our results provide the accurate conclusions. For future applications of this survey, we recommended that the survey be distributed annually at a consistent time in order to minimize seasonal externalities. Additionally, in order to maximize the generalizability of our results, careful attention was paid to provide the survey to staff members from all shifts and areas to be as inclusive and representative of the overall hospital and health center staff population as possible.

Our findings should be interpreted in light of several limitations. First, we did not examine if employee satisfaction measured by this instrument could be influenced by management interventions, but we anticipate, based on cognitive testing and management theory, that employee satisfaction could be influenced by management. Future studies are required to assess changes in employee satisfaction in response to management changes. Second, the instrument was developed specifically for the Ethiopian context and may produced biased estimates of employee satisfaction in other countries if not tested and validated first. Before we can concluded that the SEHC is a generalizable instrument, findings should be replicated in other settings to clarify that our results are not affected by cultural elements specific to Ethiopia. Additional studies should consider adaptations to ensure the instrument captures key components of employee satisfaction in local settings. Last, we had relatively few physicians who completed the survey and no staff that were employed in private sector facilities, where experiences may differ substantially. As a result, we may have underestimated the importance of specific items to staff satisfaction. Although this instrument was applicable to physicians, a physician-specific tool may allow for greater focus on issues of primary importance to physicians.

## Conclusions

The introduction of consistent and reliable measures of staff satisfaction is crucial in order to address poor employee retention rates, which threaten the successful achievement of the Millennium Development Goals in developing countries. The introduction of the SEHC survey into Ethiopian health care facilities has ample leadership support, evident by the adoption of the survey by the Ethiopian Ministry of Health into the national reformation guidelines and the presence of a SEHC overall staff satisfaction score into the key performance indicators reported by hospitals to the government on an annual basis. Such support is critical for the successful integration of staff satisfaction measures into routine healthcare facility management, leading to potential remediation of problems that reduce staff satisfaction and exacerbate excessive workforce shortages.
